# Efficiency of donepezil in elderly patients undergoing orthopaedic surgery due to underlying post-operative cognitive dysfunction: study protocol for a multicentre randomised controlled trial

**DOI:** 10.1186/s13063-021-05648-0

**Published:** 2021-10-09

**Authors:** Huichen Zhu, Lu Cong, Yi Chen, Shaoyi Chen, Lingke Chen, Zhenling Huang, Jie Zhou, Jie Xiao, Yonglei Huang, Diansan Su

**Affiliations:** Department of Anesthesiology, Renji Hospital, School of Medicine, Shanghai Jiaotong University, 160 Pujian Road, Shanghai, 200127 China

**Keywords:** Donepezil, Post-operative cognitive dysfunction, Randomised controlled trial

## Abstract

**Background:**

*Post-operative cognitive dysfunction* (POCD) is an overarching term used to describe cognitive impairment identified in the preoperative or post-operative period. After surgical operations, older patients are particularly vulnerable to memory disturbances and other types of cognitive impairment. However, the pathogenesis of POCD remains unclear with no confirmed preventable or treatable strategy available. Our previous study demonstrated that the concentration of choline acetyl transferase in the cerebral spinal fluid was a predictive factor of POCD and that donepezil, which is an acetylcholinesterase inhibitor used in clinical settings for the treatment of Alzheimer’s disease, can prevent learning and memory impairment after anaesthesia/surgery in aged mice. This study aimed to determine the critical role of donepezil in preventing cognitive impairment in elderly patients undergoing orthopaedic surgery.

**Methods:**

A multicentre, double-blind, placebo-controlled, crossover clinical trial will be performed to assess the efficacy of donepezil in elderly patients undergoing orthopaedic surgery. Participants (*n* = 360) will receive donepezil (5 mg once daily) or placebo from 1 day prior to surgery until 5 days after surgery. Neuropsychological tests will be measured at 1 day before the operation and 1 week, 1 month, 6 months and 1 year after the operation.

**Discussion:**

This research project mainly aimed to study the effects of donepezil in elderly patients undergoing orthopaedic surgery due to underlying POCD and to investigate the underlying physiological and neurobiological mechanisms of these effects. The results may provide important implications for the development of effective interfering strategies, specifically regarding cognitive dysfunction therapy using drugs.

**Trial registration:**

ClinicalTrials.govNCT04423276. Registered on 14 June 2020

**Supplementary Information:**

The online version contains supplementary material available at 10.1186/s13063-021-05648-0.

## Background

Post-operative cognitive dysfunction (POCD) has been described as an objectively measurable decline in cognitive function at varying intervals after anaesthesia and surgery, up to 3 months to 7.5 years after surgery [1, 2]. Severely affected individuals may experience personality changes and a reduced capacity for social interaction. Some patients even develop irreversible cognitive impairment [3]. Monk et al. reported that POCD leads to increased mortality in the first year after surgery [4]. Steinmetz et al. not only confirmed the results by Monk et al. but also showed that POCD after noncardiac surgery is highly associated with the risk of leaving the labour force prematurely [5]. The incidence of POCD in patients older than 60 years at discharge has been reported to be 41.4% [6]. The incidence of POCD at 7 days after lower extremity joint replacement is as high as 41–75% [7]. As a serious post-operative complication, POCD can lead to a longer bed time of patients and increase the incidence of pressure ulcers, severe pulmonary infection and lower extremity deep vein thrombosis. At the same time, patients are not conscious and cannot effectively cooperate with examination and treatment in the hospital, and post-operative rehabilitation is also greatly reduced, even leading to surgery failure. In addition, if POCD is not alleviated for a long time, hospitalisation time and cost greatly increases, and it may be life-threatening in severe cases [8–10]. However, the aetiology and mechanism of POCD remain elusive.

The cholinergic system of the central nervous system is widely considered to be essential in learning and memory, including the modulation of the acquisition, encoding, consolidation, reconsolidation, extinction and the retrieval of memory [11]. This system degenerates during ageing and in neurodegenerative diseases such as Alzheimer’s disease (AD) [11, 12]. In the normal human hippocampus, a striking and highly significant age-related decline in choline acetyltransferase (ChAT) occurs from middle to old age (between 40 and 100 years) [12]. Cholinesterase inhibitors are now commonly used to improve cognitive function in patients with AD and related dementias by enhancing central cholinergic transmission [13]. Donepezil is a highly selective and reversible acetylcholinesterase inhibitor that improves cognition and memory of mild to moderate AD in patients by increasing the concentration of acetylcholine at the neuronal synaptic cleft [14–17]. Our previous animal study demonstrated that donepezil administration prevents isoflurane-induced learning and memory impairment in aged mice [18, 19], and our clinical study showed that lower preoperative CSF levels of ChAT and ACh can predict POCD occurrence at 7 days in aged patients [20]. In a pilot study, Doraiswamy demonstrated that donepezil improved some aspects of memory in terms of cognitive decline after coronary artery bypass [21]. Therefore, a randomised controlled trial is indicated to assess the efficacy of donepezil with regard to reducing the occurrence rate of POCD among elderly patients undergoing orthopaedic surgery.

## Aims and objectives

We aim to investigate the effect of donepezil on the improvement of cognitive function in elderly patients undergoing orthopaedic surgery. Our primary objective is to evaluate the incidence of POCD 7 days (or before leaving hospital) after surgery. Our secondary objective is to evaluate the incidence of POCD at 1 month, 6 months and 1 year after surgery and post-operative delirium during the hospital stay. We predict that the donepezil intervention will alleviate post-operative cognitive impairment in elderly patients undergoing orthopaedic surgery. We aim to provide reliable clinical evidence for reducing the rate of POCD.

## Trial design

This is a protocol for a multicentre, double-blind, randomised, two-arm parallel-group, placebo-controlled, interventional clinical study. We will observe the effects of the perioperative administration of donepezil on the post-operative cognitive function of elderly patients undergoing orthopaedic surgery.

This study was presented according to the recommendations of the Standard Protocol Items: Recommendations for Interventional Trials (SPIRIT) (supplemental file, SPIRIT checklist). The study was registered on the ClinicalTrials.gov (No. NCT04423276) on 14 June 2020. The trial registration data set is presented in supplemental table.

## Methods

### Participants, interventions and outcomes

#### Study setting

This study will enrol approximately 360 participants from the Renji Hospital Shanghai Jiao Tong University School of Medicine, Tenth People’s Hospital of Tongji University and Shanghai Guanghua Hospital of Integrated Traditional Chinese and Western Medicine. This clinical trial has been approved and supported by the ethics committee of the Renji Hospital Shanghai Jiao Tong University School of Medicine (RJ17189).

#### Eligibility criteria

Patients will be recruited primarily from three centres. Table 1 presents a summary of the inclusion and exclusion criteria.

**Table 1:** Inclusion/exclusion criteria

Inclusion criteria	Exclusion criteria
1) Elder than 60 years old 2) Speak Chinese Mandarin 3) Scheduled to undergo hip or knee replacement surgery 4) The operation time is more than 2 h 5) Signed the inform consent 6) American Society of Anesthesiologists (ASA) classification I–II	1) Existing cerebral disease, or have a history of neurological and psychiatric diseases including Alzheimer Disease, stroke, epilepsy and psychosis 2) Existing cognitive impairment as evidenced by Mini-Mental State Examination scores below 24 3) Several audition or vision disorder 4) Patients with tumours or infections 5) Unwillingness to comply with the protocol or procedures 6) Cannot communicate normally in Mandarin Chinese 7) Existing bradycardiac arrhythmia (Heart rate <60 bpm for any reasons) 8) Existing gastrointestinal ulcer 9) Existing urinary incontinence 10) Existing asthma or chronic obstructive pulmonary disease 11) Post-operative admission to ICU 12) Allergic to donepezil

#### Recruitment and informed consent

**Fig. 1:**
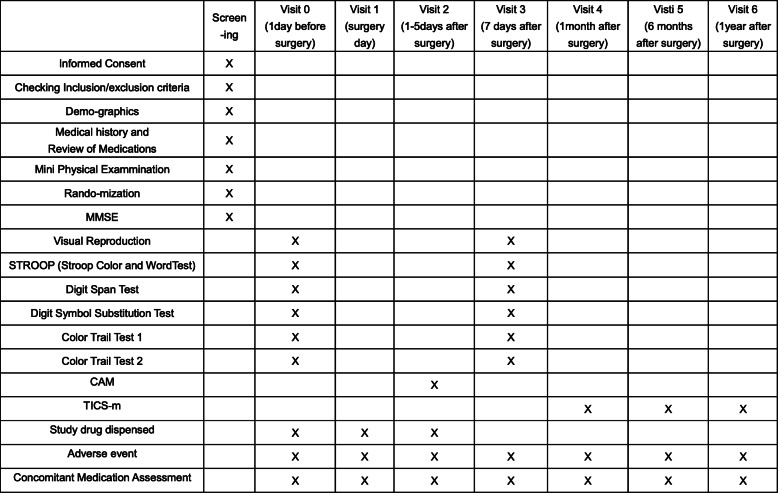
Schedule of the major study events

Figure 1 presents the schedule of the major study events for each study visit. Elderly patients who are scheduled to undergo orthopaedic surgery will be included in this study. This study is approved by the Ethics Committee of Ren Ji Hospital, Shanghai Jiao Tong University School of Medicine. Surgery will be performed within 2 days after the screening process. Designated doctors will explain this trial to interested potential participants in detail and provide them with the informed consent form. Participants are given at least 24h to decide whether they wish to participate in this trial. The informed consent form will be signed by the participant or his/her trustee or guardian and may be withdrawn at any time during the trial. Written informed consent and the patient’s baseline data will be obtained before randomisation. Moreover, participants are encouraged to contact the research team if they have any health concerns during the trial. Recruitment and consenting of study participants by the members of the research team is in-line with good clinical practice (GCP). During the clinical trial, the researchers will immediately report any serious adverse events occurring in the subjects, whether related to the drug under study or not, to the director in charge of the clinical trial of the research institution and contact Professor Diansan Su or Dr. Huichen Zhu.

#### Allocation

Subjects will be randomly assigned to one of the following two groups: donepezil group or control group (placebo). Randomisation will be performed as a block randomisation, stratified by the centre with 1:1 allocation, using SAS software. A random code will be generated between the donepezil group and the control group from each centre, in a 1:1 ratio. Envelopes containing the random codes will be generated and distributed to each centre. Drugs will be allocated according to these random numbers by personnel who are unrelated to the trial, and a code will be assigned to each drug. Upon entering the study, each subject will be assigned to either the donepezil group or the control group with an equal probability according to the random code at each centre.

#### Intervention

Subjects will receive 5 mg of donepezil or placebo once every night starting from 1 day before surgery until 5 days after surgery. On the day of surgery, patients will have a peripheral vein opened as a routine procedure. After measuring pulse oximetry, electrocardiogram and noninvasive blood pressure, general anaesthesia will be induced with 0.05 mg/kg midazolam, 1 mg/kg propofol, 0.6 mg/kg rocuronium and 0.5 μg/kg sufentanyl. Anaesthesia will be maintained with intravenous cisatracurium, sevoflurane, remifentanil and propofol. The drug dosage will be adjusted according to the patients’ hemodynamic response to maintain stable haemodynamics. To ensure that the patient’s haemoglobin level remains > 8 g/dL, blood will be transfused when the haemoglobin level drops to < 8 g/dL. Succinylcholine will be avoided during surgery. Post-operative analgesia will be given through patient-controlled intravenous analgesia. Patients will be returned to the general ward once they recover from anaesthesia.

### Interventions, modifications, adherence and concomitant care

The assigned intervention will be discontinued only in response to participant request. No modification of the interventions is planned during the trial. Adherence to interventions mainly refers to patient self-management adherence. No concomitant care or interventions are permitted during this trial.

### Plans for collection, laboratory evaluation and storage of biological specimens for genetic or molecular analysis in this trial/future use

We have no plans to collect or store biological specimens in this trial.

### Outcomes

#### Primary outcome measures

Primary outcome of this study is the incidence of POCD in the two groups during 7 days after surgery (or before leaving hospital), which was evaluated by *Z*-score (detail in the “Statistical analysis” section).

#### Secondary outcome measures

Secondary outcomes comprise the following:

1. POCD incidence in the two groups 1 month after surgery, which was evaluated by Telephone Interview for Cognitive Status—modified (TICS-m).
2. POCD incidence in the two groups 6 months after surgery, which was evaluated by TICS-m.
3. POCD incidence in the two groups 1 year after surgery, which was evaluated by TICS-m.
4. Incidence of post-operative delirium in the two groups after surgery, which was evaluated by Confusion Assessment Method (CAM).

### Participant timeline

Figure 1 presents the schedule for enrolment, interventions, assessments and visits for participants.

Cognitive function will be assessed 1 day before surgery, in the first 7 days or before leaving hospital and at 1 month, 6 months and 1 year after surgery.

The evaluation of cognitive function will also include Visual Reproduction, Stroop Colour and Word Test (STROOP), Digit Span Test, Digit Symbol Substitution Test, Colour Trail Test 1 and Colour Trail Test 2.

Post-operative delirium will be assessed using the Confusion Assessment Method (CAM). The CAM includes acute onset, inattention, disorganised thinking and abnormal consciousness. The diagnosis of delirium by the CAM requires the presence of features 1 and 2 and either 3 or 4. Table 2 presents the specific content.

**Table 2:** Specific content of the Confusion Assessment Method

The Confusion Assessment Method (CAM)	
Feature 1	Acute change in mental status with a fluctuating course Is there evidence of an acute change in mental status from the patient’s baseline? Did this behaviour fluctuate during the past day, that is, tend to come and go or increase and decrease in severity? This feature is usually obtained from a family member or nurse and is shown by positive responses.
Feature 2	Inattention Do the patients have difficulty focusing attention, for example, being easily distractible, or having difficulty keeping track of what was being said?
Feature 3	Disorganized thinking This feature is shown by a positive response to the following question: Was the patient’s thinking disorganized or incoherent, such as rambling or irrelevant conversation, unclear or illogical flow of ideas, or unpredictable switching from subject to subject?
Feature 4	Altered level of consciousness Overall, how would you rate this patient’s level of consciousness? (alert [normal], vigilant [hyperalert], lethargic [drowsy, easily aroused], stupor [difficult to arouse], or coma [unarousable])

The diagnosis of delirium by CAM requires the presence of features 1 and 2 and either 3 or 4

Long-term cognitive function will be assessed using a modified version of the Telephone Interview for Cognitive Status—modified (TICS-m) at 1 month, 6 months and 1 year after surgery. TICS-m has been used to screen for dementia and mild cognitive impairment. TICS-m scores range from 0 to 48, with higher scores indicating better function.

Evaluators will comprehensively assess the status of the patients according to their medical history, clinical observation, current psychiatric examination and information provided by family members, especially care providers [22].

### Sample size

In the previous study, we found that the POCD incidence in the elderly patients undergoing orthopaedic surgery is about 25% [20]. The anticipated effect size of donepezil is 50%. That means the power analysis for the primary outcome of POCD prevalence assumes a reduction from 25 to 12.5%. The results of a conventional analysis to detect differences in proportions (POCD) between the intervention and control groups, using PASS19.0, led to a total of 300 patients with a 1:1 randomisation, given a power of 1 − *β* = 0.80 and a two-side α level of 5%. Assuming a dropout rate of 10%, this results in a requirement of about 360 total patients.

### Allocation masking

This study is planned to follow a double-blinded method. In all cases, the investigator, intraoperative attending anaesthesiologist, evaluator, data analyser and patient will not be aware of the treatment allocation, as the medication will be encapsulated and provided by an independent nurse.

### Unblinding procedures

In the event of a medical emergency, which requires identification of an individual patient’s treatment, the investigators will be permitted to open the respective emergency envelope. A justification must be documented in the patient’s medical record and in the case report form (CRF). Serious adverse events will be collected and recorded for further analysis at the end of the trial, and investigators will explore the correlation between these events and patients’ healthcare records.

### Plans for communicating important protocol modifications to relevant parties

All changes to the study protocol will be reviewed by the ethical committee, which will be reported to the sponsor, participating care providers and investigators.

### Composition of the data-monitoring committee, its role and reporting structure

We have not considered there to be a need for a data-monitoring committee.

### Interim analyses

No formal interim analysis of the primary and secondary outcomes is planned.

### Frequency and plans for auditing trial conduct

We have no plans for auditing trial conduct in this investigator-initiated pragmatic trial.

### Data management

All patient data collected during this clinical study will be entered and/or filed in the respective patient’s CRF. The patient’s study participation must be documented appropriately in the patient CRF with study number, subject number, date of subject information and informed consent and date of each visit. Source data should be filed according to the GCP guidelines. The data manager will be responsible for data processing, according to the sponsor’s standard operating procedures. and will conduct regular monitoring to ensure that the dates are adequate, accurate and complete. Database lock will occur only after the completion of quality assurance procedures.

### Statistical analysis

Statistical analysis will be conducted using SPSS 20.0 (IBM Inc., Armonk, NY, USA). Continuous variables will be analysed using the unpaired *t* test or Mann–Whitney *U* test. Categorical variables will be analysed using the *χ*^2^ test, continuity correction *χ*^2^ test or Fisher exact test. The definition of POCD is determined using the *Z* score recommended by the International Study of Post-operative Cognitive Dysfunction studies [23]. We will compare the patients’ preoperative with post-operative scores 1 week later and divide the result by the standard deviation of the preoperative score of all patients to obtain a *Z* score for each individual test. Patients will be regarded as developing POCD if the *Z* score is ≥ 1.96 on ≥ 2 individual cognitive tests. A *P* value of < 0.05 will be considered statistically significant. The statistical code generated during the current study will be available from the corresponding author on reasonable request after the trial is completed.

### Methods in analysis to handle protocol non-adherence and any statistical methods to handle missing data

The statistical analysis will be conducted on an intention-to-treat (ITT) basis. The analyses of the outcomes will be analysed as randomised, regardless of protocol adherence. All variables will be screened for frequency and type of missingness. Multiple imputation will be used if missingness is above 5% in any variable. In the case of missing data and imputation, complete case analysis will be performed as a sensitivity analysis.

### Composition of the coordinating centre and trial steering committee

Diansan Su (DSS), the principal investigator, is responsible for preparing and revising the protocol and disseminating any changes. Huichen Zhu (HCZ) and Lu Cong (LC) are responsible for coordinating data collection and analyses and writing the scientific manuscript. Yonglei Huang (YLH), a senior investigator, is responsible for overseeing the study design and protocol and interpretation of the findings. Lingke Chen (LKC), a statistician, is responsible for overseeing any statistical analyses. Jie Zhou (JZ) and Zhenling Huang (ZLH), clinical investigators, are responsible for overseeing that the study implementation on the floor follows the protocol.

### Dissemination plans

The study results will be disseminated via articles published in peer-reviewed journals.

### Additional consent provisions for collection and use of participant data and biological specimens

No ancillary study is planned, and we have no plans to collect additional participant data or biological specimens outside of what is mentioned in this protocol.

### Adverse event reporting and harms

Donepezil, a first-line drug for AD, may cause some minor adverse effects but is generally well tolerated. We will evaluate any harm from the intervention and there is also a commentary section in the study-specific CRFs where researchers can report allocation violations or any unexpected side-effects from allocated intervention. We will collect all the expected and unexpected drug-related adverse events non-systematically and report all the harms in trial publications.

### Provisions for posttrial care

If participants are injured due to this study, in case of damage related to clinical research, they will get free treatment.

## Discussion

POCD is generally believed to indicate the degeneration of the central nervous system and the decline of nervous system function induced or aggravated by anaesthesia, surgery and other factors. A number of animal studies have shown that surgery has different effects on the cognitive function of animals of different ages [24, 25]. Our previous studies have also shown that adult mice and aged mice respond differently to isoflurane anaesthesia. The learning and memory ability of adult mice can be improved under repeated isoflurane anaesthesia [26], but the opposite is true for elderly mice. After isoflurane anaesthesia, learning and memory ability were shown to decrease significantly [18, 19]. We noticed that the central cholinergic state is different between adult mice and elderly mice and that the number of central cholinergic nerves in elderly mice decreased significantly. Before the age of 40 years, the activity of the central cholinergic system is continuously enhanced in healthy individuals, but it gradually degenerates after 40 years of age until the end of life [12]. The central cholinergic nervous system plays an important role in learning and memory as well as against central nociceptive responses. Studies have shown that nicotine or cholinesterase inhibitors can counteract cognitive impairment caused by AD [27, 28]. Additionally, in vitro studies have shown that nicotine can antagonise the neurotoxicity mediated by N-methyl-D-aspartate and β-amyloid protein [29]. Moreover, nicotine can reduce neurologic damage, behavioural changes and brain injury caused by stroke, Parkinson‘s disease in learning and memory [30–33]. The degeneration of the central cholinergic system that results from ageing is an important cause of cognitive dysfunction in elderly animals.

Donepezil is a specific acetylcholinesterase inhibitor that is widely used for the treatment of mild to moderate AD. It can improve cognitive and memory functions by increasing the concentration of acetylcholine in the synaptic spaces of neurons and slow the rate of AD progression. Donepezil has been shown to slow the progression of hippocampal atrophy in patients with AD as compared with untreated patients [34–36]. In addition to inhibiting acetylcholinesterase hydrolysis of acetylcholine, donepezil also antagonises the inhibition of N-methyl-D-aspartate receptors in the brain, improves blood circulation and reduces the neurotoxic effects of β-amyloid. It has been used in the clinical setting for the treatment of cognitive dysfunction caused by craniocerebral injury, senile vascular dementia and other diseases [34, 37]. Our previous research has found that donepezil prevent the learning and memory impairment after isoflurane exposure in aged mice [18].

In a previous study, test results from the normative population revealed that four tests were highly correlated with both age and IQ: Letter-Digit Coding, Stroop Colour Word Test, Concept Shifting Task and Visual Verbal Learning. Additionally, high test–retest reliability coefficients were obtained. Consequently, these tests were chosen to evaluate cognitive function [23]. Using this cognitive test battery, our previous study evaluated the association between the biomarker in the cerebral spinal fluid and cognitive function after surgery [20]. Our present study will also use the *Z* score to define POCD. The advantage of the *Z* score method is that it reflects subtle changes in test scores.

This will be the first multicentre randomised controlled trial to examine the effect of perioperative administration of donepezil on post-operative cognitive impairment in elderly patients after orthopaedic surgery. Our previous studies have shown that the administration of donepezil in elderly mice reduces perioperative ChAT protein expression, leading to improved cognitive function in the perioperative period [18]. We predict that the donepezil intervention will alleviate post-operative cognitive impairment in elderly patients undergoing orthopaedic surgery. We aim to provide reliable clinical evidence for reducing the rate of post-operative cognitive impairment.

### Trial status

Trial registration: ClinicalTrials.gov, NCT04423276. Registered on 14 June 2020.

The protocol version is 1.1, which was approved in January 2020. This study started in June 2020, and the recruitment phase will last until December 2021.

## Supplementary Information

**Additional file 1:.** SPIRIT checklist

**Additional file 2:.** Supplemental table

## Data Availability

The participant-level data set cannot be made publicly available because of Chinese data protection rules and regulations. The statistical code is available upon request.

## Abbreviations

POCD: Post-operative cognitive dysfunction
AD: Alzheimer’s disease
ChAT: Choline acetyltransferase
GCP: Good clinical practice
STROOP: Stroop colour and word test
CAM: Confusion assessment method
TICS-m: Telephone interview for cognitive status—modified
CRF: Case report form
