# Supplementary material for: Efficiency of donepezil in elderly patients undergoing orthopaedic surgery due to underlying post-operative cognitive dysfunction: study protocol for a multicentre randomised controlled trial
Source: Trials. 2021 Oct 9;22:688. doi: 10.1186/s13063-021-05648-0 (PMC8501596; doi:10.1186/s13063-021-05648-0)
Supplement: Supplementary file 2 — Additional file 2:. Supplemental table [file 13063_2021_5648_MOESM2_ESM.docx]

| 1. Primary registry and trial-identifying number: ClinicalTrials.gov, NCT04423276 2. Date of registration in primary registry: 14 June 2020 3. Secondary indentifying numbers: no 4. Sources of monetary or material support: no 5. Primary sponsor: no 6. Secondary sponsor(s): no 7. Contace for public queries: MD Huichen Zhu, department of anesthesiology, Renji Hospital, Shanghai Jiaotong University School of Medicine 8. Contace for scientific queries: PHD. Diansan Su, department of anesthesiology, Renji Hospital, Shanghai Jiaotong University School of Medicine 9. Publice title: Efficiency of donepezil in elderly patients undergoing orthopaedic surgery due to underlying post-operative cognitive dysfunction 10. Scientific title: see 9 11. Countries of recruitment: China, Shanghai 12. Health condition(s) or problem(s) studied: Post-operative cognitive dysfunction (POCD) 13. Intervention(s): perioperative intervention for elective orthopaedic surgery 14. Key inclusion criteria: elder than 60 years old, speak Chinese Mandarin, scheduled to undergo hip or knee replacement surgery, the operation time is more than 2 hours, signed the inform consent, ASA classification I-II   Key exclusion criteria: existing cerebral disease, or have a history of neurological and psychiatric diseases including Alzheimer Disease, stroke, epilepsy and psychosis, existing cognitive impairment as evidenced by Mini-Mental State Examination scores below 24, several audition or vision disorder, unwillingness to comply with the protocol or procedures, can not communicate normally in Mandarin Chinese,, postoperative admission to ICU, allergic to donepezil   1. Study type: multicentre randomised controlled trial 2. Date of first enrolment: 22 June 2020 3. Target sample size: 360 4. Recruitment status: enrolling by invitation 5. Primary outcome(s): the incidence of POCD 7 days after surgery (or before leaving hospital) 6. Key secondary outcome(s): POCD incidence 1 month after surgery, POCD incidence 6 months after surgery, POCD incidence 1 year after surgery, incidence of post-operative delirium after surgery |
| --- |

Supplemental Table：Items from the World Health Organizaion Trial Registration Data Set
